# Time-Dependent Differences in the Human Milk Proteome After Preterm Birth: A Paired Two-Stage Proteomic Study

**DOI:** 10.3390/nu18050848

**Published:** 2026-03-05

**Authors:** Nina Mól, Magdalena Zasada, Maciej Suski, Wojciech Zasada, Przemko Kwinta

**Affiliations:** 1 Department of Pediatrics, Jagiellonian University Medical College, Wielicka 265 Street, 30-663 Krakow, Poland; nina.mol@uj.edu.pl (N.M.); magdalena.zasada@uj.edu.pl (M.Z.); 2 Department of Pharmacology, Faculty of Medicine, Jagiellonian University Medical College, Grzegórzecka 16 Street, 31-531 Krakow, Poland; maciej.suski@uj.edu.pl; 3 Proteomics Laboratory, Centre for the Development of Therapies for Civilization and Age-Related Diseases CDT-CARD, Jagiellonian University Medical College, Skawińska 8 Street, 31-066 Krakow, Poland; 4 Clinical Department of Cardiology and Cardiovascular Interventions, University Hospital, Jakubowskiego 2 Street, 30-688 Krakow, Poland; zasada.wojciech@gmail.com; 5 Kraków Clinical Research Institute, Wadowicka 7 Street, 30-347 Krakow, Poland

**Keywords:** human milk, preterm birth, milk proteome, lactation stage, immune-related proteins, data-independent acquisition (DIA), quantitative proteomics, preterm vs. term infants

## Abstract

**Background/Objectives:** Human milk composition is shaped by gestational age at delivery and stage of lactation; however, proteomic differences between milk from mothers of preterm and term infants and their temporal patterns remain incompletely characterised. **Methods:** This prospective study enrolled 40 lactating mothers: 20 who delivered preterm infants (<32 weeks’ gestation) and 20 who delivered at term (37–42 weeks). Each provided milk samples during early lactation (first 10 days postpartum) and during later lactation (week five postpartum). Milk serum was analysed using quantitative data-independent acquisition mass spectrometry. Differential protein abundance was assessed separately at each time point; functional annotation was performed using Gene Ontology biological process analysis. **Results:** Eighty samples were analysed. On average, a total of 662 proteins were identified per sample, of which 169 were consistently quantified across all samples (1% FDR). During early lactation, 10 proteins differed significantly, with bidirectional changes and moderate effect sizes. At week five, 19 proteins were differentially abundant, predominantly higher in preterm samples. Immune-related proteins constituted the largest functional category at both stages. Immunoglobulin heavy constant gamma 4 remained consistently downregulated in preterm milk (1.6-fold lower abundance). Ferritin heavy chain (1.5) and HLA class II histocompatibility antigen gamma chain (1.8) were elevated only early, whereas calprotectin subunits S100A8 (5.6) and S100A9 (5.2) were markedly upregulated later. **Conclusions:** Proteomic differences vary across lactation stages, highlighting lactation stage as an essential contextual variable in comparative milk proteomics.

## 1. Introduction

Preterm birth is associated with increased nutritional vulnerability resulting from the immaturity of metabolic, gastrointestinal, and immune systems. Optimal postnatal nutrition is therefore a critical component of early care in preterm infants, with human milk consistently recognized as the preferred source of enteral feeding due to its unique nutritional and bioactive properties [1].

Beyond its macronutrient content, human milk contains a complex and dynamic array of proteins involved in immune regulation, inflammatory signaling, tissue development, and host defense [2,3]. The composition of human milk is not static but changes across the course of lactation, reflecting both physiological maturation and adaptive responses to infant needs. These temporal changes are particularly relevant in the context of preterm delivery, where early postnatal requirements differ substantially from those of term infants [4].

Previous studies have reported compositional differences between milk from mothers of preterm and term infants, especially during early lactation. However, most investigations have focused on selected proteins or predefined immunological components, providing limited insight into coordinated changes within the broader milk proteome.

However, it remains unclear whether preterm delivery is associated with a stable, delivery-dependent proteomic signature or with stage-specific quantitative modulation across lactation [5].

High-throughput proteomic approaches offer the opportunity to comprehensively characterize the milk proteome and to detect subtle but biologically coherent changes in protein abundance that may not be captured by targeted analyses. Quantitative proteomic approaches based on data-independent acquisition enable reproducible profiling of complex biological fluids, including human milk, and are well suited for assessing time-dependent changes across lactation [6,7,8,9]. The data-independent acquisition (DIA) strategy has recently emerged as a new mass spectrometric technique that aims to merge the comprehensive coverage of shotgun proteomics with the reproducibility and accuracy of selected reaction monitoring. Bruderer et al. [10] demonstrated in a model study that DIA surpasses shotgun (DDA) proteomics in both the number of peptides consistently identified across repeated measurements and in the quantification of differentially abundant proteins. The reproducibility of peptide detection using DIA was more than twice as high as that achieved with shotgun proteomics, indicating that DIA should be considered the method of choice for quantitative protein profiling. Despite this potential, comparative proteomic studies that simultaneously address gestational age at delivery and changes across lactation remain scarce. In parallel, recent proteomic and multi-omics studies have demonstrated substantial interindividual variability and temporal dynamics in human milk composition [7], underscoring the need for carefully designed comparative analyses.

Understanding the temporal dynamics of the human milk proteome in mothers of preterm infants is essential for interpreting observed compositional differences within a nutritional framework and for distinguishing early, transient adaptations from more persistent features of milk composition. Such knowledge is particularly relevant in clinical nutrition, where human milk is increasingly viewed as an active, adaptive component of neonatal nutritional support rather than a uniform dietary substrate.

Therefore, the aim of this study was to perform a comparative quantitative proteomic analysis of human milk from mothers of preterm and term infants collected at two predefined stages of lactation, to assess whether proteomic differences are consistent or stage-specific.

## 2. Materials and Methods

### 2.1. Study Population

The study prospectively included 40 lactating mothers whose infants were admitted to the Neonatal Intensive Care Unit of the Department of Paediatrics, Jagiellonian University Medical College, Krakow, Poland, between October 2020 and November 2021. Two groups were enrolled: mothers of preterm infants born before 32 weeks of gestation and mothers of term infants born between 37 and 42 weeks of gestation. Exclusion criteria for both groups included maternal diabetes mellitus (gestational or pregestational), neonatal phenylketonuria, and other severe maternal diseases potentially affecting lactation, as assessed by the investigators. Each participating mother provided two paired milk samples, collected at two predefined time points: early lactation (first 10 days postpartum) and later lactation (week five postpartum). Maternal anthropometric data, including height and pregnancy-related weight measures, were recorded. Maternal diet was not systematically assessed. Postpartum systemic glucocorticoid therapy was not reported among participants. Although selected perinatal and maternal clinical variables were documented, adjustment for these factors in proteomic analyses was not feasible due to limited sample size and the high dimensionality of the dataset.

### 2.2. Sample Preparation

#### 2.2.1. Milk Collection

All milk samples were collected at noon after a minimum fasting period of four hours to minimize short-term dietary variability, although proteomic composition is not expected to exhibit major acute postprandial fluctuations. Milk expression was performed using an electric breast pump (Medela AG, Baar, Switzerland) with disposable Symphony one-day pump sets for a duration of 10 min. From each expressed sample, 5 mL of milk was collected and immediately stored at −80 °C until further analysis.

#### 2.2.2. Milk Serum Separation

To minimize interference from milk fat globules and casein micelles during proteomic analysis, milk serum was isolated prior to protein extraction. Samples were thawed at room temperature and centrifuged at 1500× *g* for 10 min to remove the lipid layer. The resulting supernatant was subsequently ultracentrifuged at 100,000× *g* for 90 min at 4 °C using a Beckman L-60 ultracentrifuge. The clarified milk serum fraction was collected and used for further proteomic analysis.

#### 2.2.3. Sample Preparation for LC-MS/MS Analysis

Milk samples (20 μL) were mixed with 80 µL of lysis buffer (2.5% SDS, 60 mM DTT in 0.1 M Tris-HCl pH 7.6), vortexed, incubated at 95 °C for 5 min and clarified by centrifugation at 14,000× *g* for 30 min. Before protein digestion the total protein concentration in collected lysates was determined by WF-assay [11]. Next, a volume containing 70 µg of total protein was transferred to Microcon-30 kDa centrifugal filter units (Merck, Darmstadt, Germany), denatured with 8 M urea in 0.1 M Tris-HCl pH 8.5 and digested to peptides with a use of filter-aided sample preparation (FASP) protocol [12]. Briefly, proteins were alkylated with iodoacetamide and cleaved with LysC-trypsin mix (Thermo Scientific, Waltham, MA, USA) with an enzyme-to-protein ratio of 1:50. Digestions were carried out overnight in 50 mM Tris-HCl pH 8.5 at 37 °C. After digestion the peptide yields were determined by WF-assay and the aliquots containing equal amount of total peptides were desalted on 96-Well MiniSpin C18 columns (Harvard Apparatus, Holliston, MA, USA). Samples were then concentrated to a volume of ~5 µL and stored at −80 °C. For project-specific spectral libraries preparation equal amount of peptides from all samples were combined and subjected to fractionation protocol. HpH fractionation on C18 Micro SpinColumns (Harvard Apparatus, Holliston, MA, USA) was performed in 50 mM ammonium formate buffer (pH 10) with 13 consecutive injections of the eluent buffer, comprising 5, 10, 12.5, 15, 17.5, 20, 22.5, 25, 27.5, 30, 35 and 50% acetonitrile in 50 mM ammonium formate buffer (pH 10), collected by centrifugation (300× *g*, 2 min) and dried in a speedvac concentrator (Eppendorf, Hamburg, Germany). In this way peptides were distributed across 12 HpH fractions and analyzed by LC-MS/MS in DDA acquisition mode for library generation. Prior the analysis all samples and library peptide fractions were solubilized in 0.1% formic acid in a concentration of 0.5 µg/µL and spiked with the iRT peptide mix (Biognosys, Schlieren, Switzerland) for normalization of the retention time.

#### 2.2.4. Liquid Chromatography–Tandem Mass Spectrometry

Peptides (1 µg) were injected onto a nanoEase M/Z Peptide BEH C18 75 µm i.d. × 25 cm column (Waters, Milford, MA) via a trap column nanoEase M/Z Symmetry C18 180 µm i.d. × 2 cm column (Waters, Milford, MA, USA). For library generation, each peptide fraction was separated using a 98 min 1% to 40% B phase linear gradient (A phase—0.1% FA; B phase—80% ACN and 0.1% FA) operating at a flow rate of 300 nL/min on an UltiMate 3000 HPLC system (Thermo Scientific, Waltham, MA, USA) and applied to a TripleTOF 6600+ (Sciex, Framingham, MA, USA) mass spectrometer. The main working nano-electrospray ion source (Optiflow, Sciex, Framingham, MA, USA) parameters were as follows: ion spray voltage 3.2 kV, interface heater temperature (IHT) 200 °C, ion source gas 1 (GS1) 10 and curtain gas (CUR) 25. For DDA acquisition, spectra were collected in full scan mode (350–1400 Da), followed by one hundred CID MS/MS scans of one hundred most intense precursor ions from the preceding survey full scan exceeding 100 cps intensity under dynamic exclusion criteria. Samples analyzed in DIA mode were separated using a 63 min 1% to 40% B phase linear gradient at a flow rate of 300 nL/min. For DIA acquisition, spectra were collected in full scan mode (400–1250 Da), followed by one hundred DIA MS/MS scans using a variable precursor isolation window approach, with *m*/*z* windows ranging from 6 to 90 Da.

#### 2.2.5. Mass Spectrometric Raw Data Analysis, Spectral Library Generation and DIA Quantitation

DDA data were searched against the human UniProt database (release 2021_01_04, 17,056 entries) using the Pulsar search engine implemented in Spectronaut software (Biognosys, Schlieren, Switzerland) [10] with default parameters (±40 ppm mass tolerance on MS1 and MS2 level, mutated decoy generation method, trypsin enzyme specificity, version 18). Deep Learning Assisted iRT Regression was set as iRT reference strategy for RT to iRT calibration with minimum R2 set to 0.8. Peptide, protein and PSM FDR were set to 1%. Library was generated using 3–6 fragment ions per precursor.

The project-specific library was be then used to analyze the DIA data in Spectronaut (Biognosys, Schlieren, Switzerland). Data were filtered by 1% FDR on peptide and protein level, while quantitation and interference correction were done on the MS2 level. Protein grouping was performed based on the ID picker algorithm [13]. Protein quantities were calculated by averaging the respective peptide intensities, while the latter were obtained as mean precursor quantities. Two-sample *t*-tests were performed with false discovery rate (FDR) correction using the Storey method [14]. Proteins with *q* < 0.05 were considered statistically significant. The Partial Least Squares Discriminant Analysis (PLS-DA) was done in RStudio 2026.01.0 (Build 392) with the use of mixOmics package [15].

### 2.3. Statistical Analysis

Categorical variables are presented as numbers and percentages. Continuous variables are expressed as mean and standard deviation (SD) or as median with first and third quartiles (Q1–Q3), as appropriate. Normality of distribution was assessed using the Shapiro–Wilk test.

Differences between groups were analyzed using Student’s *t*-test for normally distributed variables and the Wilcoxon test for non-normally distributed variables. Paired analyses were applied where appropriate to account for the longitudinal, within-subject design. Because comparisons between preterm and term milk were conducted separately at each lactation stage, analyses were performed using unpaired group comparisons at each time point. Pairing refers to sample structure, not time-trend testing. Categorical variables were compared using Pearson’s chi-squared test or Fisher’s exact test when the assumptions of the chi-squared test were not met. Adjustment for potential confounders (including antenatal corticosteroid exposure and mode of delivery) was considered. However, given the limited sample size and the high dimensionality of the proteomic dataset, multivariable modelling was not feasible without substantial risk of model overfitting and unstable estimates. Therefore, differential protein abundance analyses were performed as predefined stage-specific comparisons without covariate adjustment.

Differential protein abundance analyses were conducted separately for each lactation time point. Proteins were considered significantly different between groups if they met the predefined statistical threshold (*q*-value < 0.05) and an absolute fold change value greater than 1.5, as reported in the corresponding result tables. An absolute fold-change (|FC|) threshold of 1.5 was applied to focus on biologically meaningful differences and reduce overinterpretation of marginal quantitative shifts. Only proteins quantified with at least two unique peptides were retained for statistical analysis. Samples were analyzed in randomized order. Quality control samples (1 μg of HeLa digest, Thermo Scientific) were injected before and after sample batch analysis to monitor instrument stability. Instrument performance and the quality of chromatographic separations were monitored using iRT peptides.

## 3. Results

### 3.1. Study Population and Maternal Characteristics

Milk samples were collected from 40 lactating mothers of enrolled infants, including 20 mothers of preterm infants (group A) and 20 mothers of term infants (group B). Baseline maternal, pregnancy, and neonatal characteristics are summarized in Table 1.

No significant differences were observed between mothers of preterm and term infants with respect to gravidity, parity, maternal age, height, educational level, prevalence of allergic diseases, smoking status, alcohol use, or previous breastfeeding history. Maternal weight gain during pregnancy was significantly lower in mothers of preterm infants compared with mothers of term infants (median 9.5 vs. 15.0 kg, *p* = 0.0003). Maternal weight gain differed significantly between groups and may represent a potential confounding factor. No significant differences were found in maternal maximal weight before delivery or maternal weight one week postpartum. No cases of clinical mastitis were reported during sampling.

Pregnancy-related conditions, including thyroid disease, hypertension, anemia, proteinuria, swelling, and bacterial or viral infections, did not differ significantly between groups. Antenatal corticosteroid prophylaxis was significantly more frequent in the preterm group (*p* = 0.0036).

As expected, gestational age and birth weight were significantly lower in the preterm group (both *p* < 0.0001). Caesarean section was more common among preterm deliveries (*p* = 0.0084). No significant differences were observed in newborn sex distribution.

### 3.2. Differential Milk Proteome in Early Lactation

To facilitate functional interpretation, differentially abundant proteins were annotated using Gene Ontology (GO) terms with the DAVID bioinformatics resource [16,17].

On average, a total of 662 proteins were identified per sample, of which 169 were consistently quantified across all samples (1% FDR).

**Figure 1:**
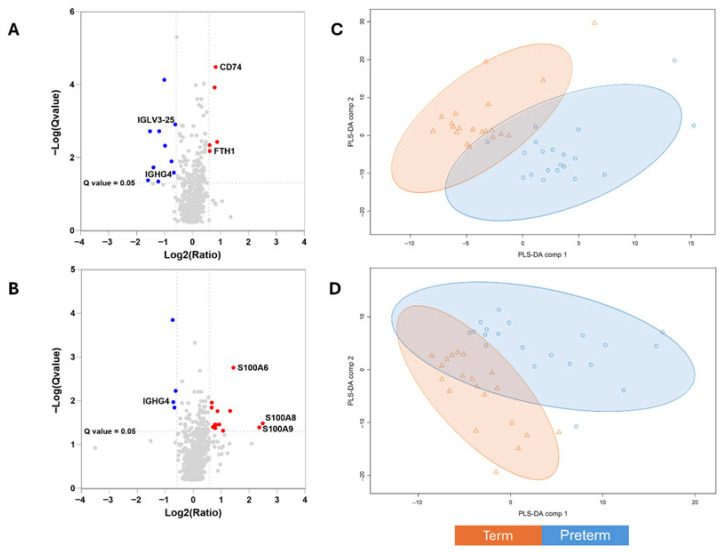
Differential proteomic profiles of human milk. Differentially abundant proteins and partial least squares discriminant analysis (PLS-DA during early lactation (**A**,**C**) and later lactation (**B**,**D**).

Global proteomic differences between milk from mothers of preterm and term infants at each lactation stage are summarized in Figure 1. Volcano plot analysis illustrates the magnitude and statistical significance of protein abundance differences during early and later lactation, while multivariate PLS-DA based on whole-proteome data demonstrates clear separation between preterm and term milk samples at both time points. Ranked protein intensities distribution obtained by DIA quantitation is presented on Supplementary Figure S1.

Quantitative proteomic analysis revealed effect sizes ranged between 1.5- and 2.3-fold in protein abundance between milk samples from mothers of preterm and term infants at two predefined stages of lactation. Volcano plots illustrate differentially abundant proteins during early lactation (A) and later lactation (B). Multivariate analysis based on whole-proteome data using partial least squares discriminant analysis (PLS-DA) demonstrated clear separation between preterm and term milk samples at both time points (C and D, respectively). Differentially abundant proteins were defined as those meeting *q*-value < 0.05 after Storey correction and absolute fold change > 1.5.

During early lactation, only a limited number of proteins differed between groups, with changes predominantly involving immune-related proteins and modest effect sizes. Specifically, quantitative proteomic analysis of milk samples collected during early lactation identified 10 proteins that differed significantly between mothers of preterm and term infants (Table 2).

Differences were bidirectional, although several immunoglobulin-related proteins showed reduced abundance in preterm milk. Proteins increased in preterm milk during early lactation were predominantly related to iron handling and immune regulation, including ferritin heavy chain and HLA class II histocompatibility antigen gamma chain, both showing approximately 1.5–1.8-fold higher abundance. In contrast, several immunoglobulin-related proteins, including immunoglobulin heavy constant gamma 4 and immunoglobulin lambda variable 3–25, were present at lower levels in preterm milk, with fold changes ranging from approximately 1.5- to 2.3-fold lower abundance.

Overall, the magnitude of early-lactation differences was moderate, and the differentially abundant proteins were mainly annotated to immune system-related biological processes, with additional representation of proteins involved in signaling and digestive pathways.

### 3.3. Differential Milk Proteome in Later Lactation

During later lactation, the number and magnitude of proteomic differences increased, with a clear predominance of higher protein abundance in preterm milk (Table 3). At this time point, the pattern of differences shifted, with most proteins exhibiting higher abundance in preterm milk.

The most pronounced changes were observed for calprotectin subunits S100A8 and S100A9, which showed more than five-fold higher abundance in preterm milk compared with term milk. Despite large fold changes, within-group variability remained substantial, consistent with known interindividual heterogeneity in milk proteomics. No extreme outliers were identified upon inspection of normalized intensity distributions.

Additional immune-related proteins, including S100A6 and multiple immunoglobulin variable region components, also demonstrated increased abundance in preterm milk, typically in the range of 1.6- to 2.7-fold. In contrast, only a small number of proteins, including immunoglobulin heavy constant gamma 4, remained lower in preterm milk at this later stage.

As in early lactation, most differentially abundant proteins identified at week five were associated with immune-related biological processes, with a smaller subset linked to signaling and structural functions.

A ranked list of proteins identified during early lactation according to absolute fold change is provided in Supplementary Table S1. A ranked list of proteins identified during later lactation according to absolute fold change is provided in Supplementary Table S2.

## 4. Discussion

### 4.1. Summary of Principal Findings

In this paired proteomic analysis of human milk from mothers delivering preterm and term infants, we identified a limited but biologically coherent set of differentially abundant proteins between groups. The proportion of differentially abundant proteins represented approximately 1.5% of the quantified proteome at early lactation and 2.8% at later lactation. The number of differentially abundant proteins differed between predefined lactation stages, with fewer proteins detected during early lactation and a higher number during later lactation. Importantly, the direction of differences between preterm and term milk was not uniform across lactation stages, suggesting stage-specific modulation rather than a stable group-defining proteomic signature. Proteins related to immune function constituted the dominant functional category among the differentially abundant proteins at both stages. Several proteins exhibited clear stage-dependent patterns, including IGHG4, ferritin heavy chain, and the calprotectin subunits S100A8 and S100A9. Accordingly, the present analyses were restricted to predefined stage-based comparisons, and the findings should be interpreted within this analytical framework.

### 4.2. Biological Interpretation of Key Proteins

#### 4.2.1. Immune-Related Proteins and Immunoglobulins

Immunoglobulin-related proteins represented a prominent component of the observed proteomic differences between preterm and term milk, consistent with the established immunological role of human milk [3,5]. Among these, IGHG4 showed a consistent decrease in preterm milk compared with term milk across lactation stages. IgG subclasses are known to differ substantially in effector function and immunological relevance, and IgG4 in particular has been associated with regulatory or tolerogenic immune responses rather than classical pro-inflammatory activity [18]. Therefore, the observed reduction in IGHG4 should not be interpreted as indicating a global impairment of immune protection but rather as a selective alteration within the immunoglobulin repertoire. Marked interindividual variability in immunoglobulin abundance has been repeatedly reported in human milk proteomic studies, even within homogeneous populations [8,18], supporting cautious interpretation of subclass-specific differences. In early lactation, the detection of HLA class II-related proteins aligns with previous observations that antigen presentation-associated components may be more prominent shortly after delivery, potentially reflecting maternal immune signaling rather than direct infant immune activation [4].

#### 4.2.2. Proteins Related to Innate Immunity and Inflammatory Response

Proteins involved in innate immune defense constituted a second major group among the differentially abundant proteins. The calprotectin complex, composed of S100A8 and S100A9, showed stage-dependent differences between preterm and term milk. Calprotectin is well recognized for its antimicrobial properties and its role in nutritional immunity through metal chelation, but it is also widely used as a biomarker of inflammatory activity [19,20]. Calprotectin, a key component of innate immune responses, has been proposed to contribute to mucosal immune protection in early life; however, evidence in the context of human milk remains indirect and largely inferential [21]. However, increased abundance may reflect maternal systemic or mammary gland inflammatory activity rather than an adaptive milk-specific mechanism, although no inflammatory markers were measured in the present study.

Rather than an adaptive milk-specific mechanism, and these interpretations are not mutually exclusive. Accordingly, the present findings are best interpreted as indicating differential representation of innate immune-related proteins rather than direct evidence of altered inflammatory signaling.

#### 4.2.3. Iron Metabolism and Adaptive Functions

Ferritin heavy chain emerged as a protein with clear stage-dependent differences between preterm and term milk. Ferritin in human milk has been proposed to play a dual role, acting both as an iron-binding protein and as a potential modulator of oxidative stress [22,23]. Previous studies have reported variability in milk ferritin concentrations across lactation and between individuals, suggesting tight regulation rather than passive diffusion from maternal circulation [22,24,25]. In the context of preterm birth, altered ferritin abundance may reflect adaptive modulation of iron handling in milk rather than differences in total iron supply. Importantly, the present study was not designed to assess iron bioavailability or clinical iron status, and no clinical inferences regarding infant iron nutrition can be drawn from these findings.

### 4.3. Lactation Stage as Contextual Framework Rather than Primary Driver

The interpretation of proteomic differences in human milk must account for the strong influence of lactation stage on milk composition. Rather than assuming that lactation stage itself drives the observed differences, our findings suggest that differences observed at predefined stages reflect temporal modulation of group-specific patterns. This distinction is important, as the direction and magnitude of differences between preterm and term milk varied between stages, arguing against a single, stable proteomic phenotype. Formal longitudinal analyses within individuals across lactation stages were not performed, and therefore no causal inferences regarding temporal changes can be made. Formal interaction testing between lactation stage and delivery group was not performed due to sample size limitations.

Similar stage-dependent variability has been reported in previous proteomic and compositional studies of human milk, underscoring the importance of contextualizing group comparisons within clearly defined lactation windows [4,9].

### 4.4. Novelty and Significance of the Study

The primary strength of this study lies in its design and analytical approach rather than in the identification of individual biomarkers. The use of paired sampling, with two samples obtained from the same mother at predefined lactation stages, minimizes interindividual variability that is known to be substantial in human milk proteomics [7]. Quantitative DIA-based proteomics enabled robust comparison at the proteome level rather than reliance on selected candidate proteins. Importantly, comparisons between preterm and term milk were conducted separately for each lactation stage, avoiding implicit assumptions of temporal continuity. Together, these design elements allow for a more nuanced interpretation of proteomic differences than is typically possible in cross-sectional milk studies.

### 4.5. Limitations

Several limitations of the present study should be acknowledged. The sample size was limited, reflecting the complexity of paired proteomic analyses in human milk. Functional validation of the identified proteins was not performed, and conclusions are restricted to differences in protein abundance. The absence of orthogonal validation (e.g., ELISA or targeted proteomics) limits confirmation of key quantitative findings.

Within-group longitudinal changes across lactation stages were not analyzed, precluding direct assessment of temporal trajectories. Mixed-effects modelling was not performed due to sample size constraints. Finally, unmeasured maternal and perinatal factors may have contributed to the observed variability. Although certain perinatal factors, including antenatal corticosteroid exposure and mode of delivery, differed between groups, adjustment was not performed due to limited sample size and the high dimensionality of proteomic data, which would have resulted in unstable multivariable models.

### 4.6. Implications and Future Directions

The present findings contribute to a growing body of evidence indicating that differences in human milk composition between preterm and term deliveries are protein-specific and context-dependent rather than uniform. These results underscore the importance of considering lactation stage and individual variability when interpreting milk proteomic data. Future studies combining longitudinal designs with functional assays will be required to clarify the biological significance of stage-dependent protein modulation. At present, the implications of these findings are primarily relevant for research into human milk biology rather than for clinical practice. Future studies integrating proteomic profiling with clinical outcome data, including gut microbiota development, intestinal barrier maturation, and the incidence of late-onset sepsis, are required to determine the biological and potential clinical significance of the observed protein-level differences.

Although stage-dependent differences in immune-related proteins were observed, they should not be interpreted as warranting changes in current neonatal feeding or fortification strategies. Instead, the results provide a mechanistic framework for future investigations into how bioactive milk components may interact with infant physiology.

Current fortification practices are primarily designed to optimize macronutrient and selected micronutrient content rather than bioactive protein composition. Whether fortification procedures influence the relative concentration or biological activity of immune-related milk proteins remains unclear and warrants dedicated investigation.

## 5. Conclusions

In conclusion, this comparative proteomic analysis demonstrates that differences between human milk from mothers of preterm and term infants are not static but vary according to the stage of lactation. Rather than revealing a single, stable proteomic phenotype associated with preterm delivery, the findings indicate stage-specific modulation of a limited set of proteins, predominantly related to immune function. Importantly, the direction and magnitude of these differences changed between early and later lactation, underscoring lactation timing as a critical contextual factor in the interpretation of human milk proteomic data. These results suggest that observed compositional differences should be viewed as dynamic and context-dependent rather than as fixed characteristics of preterm milk. While the functional implications of these protein-level differences remain to be established, the present study highlights the importance of temporally resolved, paired study designs in advancing our understanding of human milk biology and cautions against cross-sectional comparisons across heterogeneous lactation windows.

## Figures and Tables

**Table 1:** Maternal, pregnancy, and neonatal characteristics of the study population.

	Mothers of Full-Term Babies (*n* = 20)	Mothers of Preterm Babies (*n* = 20)	*p*-Value
**Characteristics of Mothers**			
Gravidity; Me (Q1–Q3)	1 (1–2)	2 (1–3)	0.1645 ^W^
Parity; Me (Q1–Q3)	1 (1–2)	2 (1–2)	0.2057 ^W^
Maternal age [years]; mean (SD)	30.2 (5.5)	29.4 (6.1)	0.7346 ^T^
Level of education; *n* (%)			
elementary	2 (10%)	1 (5%)	0.2356 ^F^
secondary	9 (45%)	14 (70%)	
university	9 (45%)	4 (20%)	
vocational	0 (0%)	1 (5%)	
Maternal height [cm]; mean (SD)	165.6 (6.0)	162.4 (5.2)	0.0839 ^T^
Maternal weight gain during pregnancy [kg]; Me (Q1–Q3)	15.0 (12.6–17.0)	9.5 (7.3–11.8)	0.0003 ^W^
Maternal maximal weight before labour [kg]; Me (Q1–Q3)	76.0 (60.8–85.8)	70.0 (55.5–75.0)	0.0933 ^W^
Maternal weight one week after labour [kg]; Me (Q1–Q3)	72 (65–84)	64 (58–85)	0.0983 ^W^
Previously breastfeeding (whole group; *n* (%)	6 (30%)	10 (50%)	0.1967 ^P^
Breastfeeding a previous child (only if there was a previous child); *n* (%)	6 (86%)	10 (91%)	0.7324 ^P^
Interval between end of previous lactation [months]; mean (SD)	74 (45)	52 (32)	0.3299 ^T^
**Characteristics of the current pregnancy**			
Thyroid disease (any); *n* (%)	5 (25%)	2 (10%)	0.2119 ^P^
Diabetes (any); *n* (%)	0 (0%)	0 (0%)	-
Heart problems; *n* (%)	0 (0%)	0 (0%)	-
Hypertension; *n* (%)	3 (15%)	4 (20%)	0.6773 ^P^
Proteinuria; *n* (%)	0 (0%)	2 (10%)	0.1468 ^P^
Swelling; *n* (%)	2 (10%)	5 (25%)	0.2119 ^P^
Anaemia; *n* (%)	4 (20%)	3 (15%)	0.6773 ^P^
Bacterial infection (any); *n* (%)	4 (20%)	4 (20%)	0.9999 ^P^
Viral infection (any); *n* (%)	6 (30%)	2 (10%)	0.1138 ^P^
Urinary tract infection; *n* (%)	3 (15%)	1 (5%)	0.2918 ^P^
Vaginal rectal screening for GBS colonization; *n* (%)			
Positive	6 (32%)	1 (7%)	0.0897 ^P^
Negative	13 (68%)	13 (93%)	
Smoking; *n* (%)	1 (5%)	2 (10%)	0.5483 ^P^
Alcohol use; *n* (%)	0 (0%)	0 (0%)	-
Antenatal corticosteroid prophylaxis; *n* (%)			
No	20 (100%)	13 (65%)	0.0036 ^P^
Yes	0 (0%)	7 (35%)	
Gestational age [weeks]; Me (Q1–Q3)	39 (38–40)	30 (29–31)	<0.0001 ^W^
Birth weight [g], Me (Q1-Q3)	3020 (2843–3515)	1400 (1225–1575)	<0.0001 ^W^
Mode of delivery; *n* (%)			
Caesarean section	11 (55%)	18 (90%)	0.0084 ^F^
Vaginal	9 (45%)	1 (5%)	
Vaginal + caesarean section (twin pregnancy)	0 (0%)	1 (5%)	
Newborn’s gender; *n* (%)			
Female	12 (60%)	7 (35%)	0.2049 ^F^
Female + Male (twin pregnancy)	0 (0%)	1 (5%)	
Male	8 (40%)	12 (60%)	

Data are presented as mean ± SD or median (Q1–Q3), as appropriate, or as number (percentage). Between-group comparisons were performed using Student’s *t*-test (T) or the Wilcoxon test (W) for continuous variables and Pearson’s chi-squared test (P) or Fisher’s exact test (F) for categorical variables, as appropriate. Normality was assessed using the Shapiro–Wilk test.

**Table 2:** Differentially abundant proteins in human milk during early lactation.

UniProt Accession	Protein Name	Preterm vs. Term (Ratio)
**Immunity**		
P02794	Ferritin heavy chain	1.5
P04233	HLA class II histocompatibility antigen gamma chain	1.8
P01717	Immunoglobulin lambda variable 3–25	0.67
P01861	Immunoglobulin heavy constant gamma 4	0.62
**Signalling process**		
P0DTE7;P0DTE8;P0DUB6	Alpha-amylase 1B;Alpha-amylase 1C;Alpha-amylase 1A	1.5
Q13444	Disintegrin and metalloproteinase domain-containing protein 15	1.8
O00300	Tumor necrosis factor receptor superfamily member 11B	0.5
Q6UX06	Olfactomedin-4	0.43
**Other biological processes**		
P10451	Osteopontin	1.7
Q10472	Polypeptide N-acetylgalactosaminyltransferase 1	0.59

Differentially abundant proteins identified in milk samples collected during early lactation from mothers of preterm and term infants. Protein abundance differences are expressed as Preterm vs. Term (fold change). Fold change values > 1 indicate higher protein abundance in milk from mothers of preterm infants, whereas values < 1 indicate lower abundance compared with term controls. Only proteins meeting the predefined statistical significance threshold (*q*-value < 0.05) are shown. Functional grouping was based on Gene Ontology biological process annotations using the DAVID bioinformatics resource; proteins may be involved in multiple biological pathways.

**Table 3:** Differentially abundant proteins in human milk during later lactation.

UniProt Accession	Protein Name	Preterm vs. Term (Ratio)
**Immunity**		
P05109	Protein S100-A8	5.6
P06702	Protein S100-A9	5.2
P00738	Haptoglobin	1.6
P06703	Protein S100-A6	2.7
P01861	Immunoglobulin heavy constant gamma 4	0.62
P01780	Immunoglobulin heavy variable 3–7	1.8
A0A075B6R9;A0A0C4DH68	Probable non-functional immunoglobulin kappa variable 2D-24; Immunoglobulin kappa variable 2–24	1.7
A0A075B6I0	Immunoglobulin lambda variable 8–61	1.9
**Signalling process**		
O00592	Podocalyxin	2.1
Q96DA0	Zymogen granule protein 16 homolog B	1.8
P04114	Apolipoprotein B-100	0.59
P12273	Prolactin-inducible protein	1.7
Q06481	Amyloid-like protein 2	1.7
P0DTE7;P0DTE8;P0DUB6	Alpha-amylase 1B; Alpha-amylase 1C; Alpha-amylase 1A	1.6
Q9UNW1	Multiple inositol polyphosphate phosphatase 1	0.67
Q6WN34	Chordin-like protein 2	0.62
**Other**		
P13796	Plastin-2	2.5
P07437	Tubulin beta chain	1.7
P16671	Platelet glycoprotein 4	1.7

Differentially abundant proteins identified in milk samples collected during later lactation from mothers of preterm and term infants. Protein abundance differences are expressed as Preterm vs. Term (fold change). Fold change values > 1 indicate higher protein abundance in milk from mothers of preterm infants, whereas values < 1 indicate lower abundance compared with term controls. Only proteins meeting the predefined statistical significance threshold (*q*-value < 0.05) are shown.

## Data Availability

The mass spectrometry proteomics data have been deposited to the ProteomeXchange Consortium via the PRIDE [26] partner repository with the dataset identifier PXD074649. Clinical and demographic data are not publicly available due to ethical and privacy restrictions.

## Supplementary Materials

The following supporting information can be downloaded at: https://www.mdpi.com/article/10.3390/nu18050848/s1, Table S1: Top 10 differentially abundant proteins in human milk during early lactation. Table S2: Top 10 differentially abundant proteins in human milk during later lactation. Figure S1: Protein intensities distribution in DIA-based human milk proteome quantitation.
