# Supplementary material for: Time-Dependent Differences in the Human Milk Proteome After Preterm Birth: A Paired Two-Stage Proteomic Study
Source: Nutrients. 2026 Mar 5;18(5):848. doi: 10.3390/nu18050848 (PMC12987234; doi:10.3390/nu18050848)
Supplement: Supplementary file 1 [file nutrients-18-00848-s001.zip › nutrients-4174763-supplementary.pdf]

Time-dependent differences in the human milk proteome after preterm birth: a paired two-stage proteomic study

Nina Mól, Magdalena Zasada, Maciej Suski, Wojciech Zasada, Przemko Kwinta

Supplementary material

Supplementary Table S1: Top 10 differentially abundant proteins in human milk during early lactation.

Protein Groups	Genes	Protein Descriptions	Qvalue	Ratio
Q13444	ADAM15	Disintegrin and metalloproteinase domain-containing protein 15	3.75E-03	1.84
P04233	CD74	HLA class II histocompatibility antigen gamma chain	3.30E-05	1.77
P10451	SPP1	Osteopontin	1.20E-04	1.73
P02794	FTH1	Ferritin heavy chain	6.68E-03	1.53
P0DTE7; P0DTE8; P0DUB6	AMY1B; AMY1C; AMY1A	Alpha-amylase 1B;Alpha-amylase 1C;Alpha-amylase 1A	4.57E-03	1.53
P01717	IGLV3-25	Immunoglobulin lambda variable 3-25	1.24E-03	0.65
P01861	IGHG4	Immunoglobulin heavy constant gamma 4	2.60E-02	0.63
Q10472	GALNT1	Polypeptide N-acetylgalactosaminyltransferase 1	1.27E-02	0.59
O00300	TNFRSF11B	Tumor necrosis factor receptor superfamily member 11B	7.43E-05	0.50
Q6UX06	OLFM4	Olfactomedin-4	4.54E-02	0.43

Supplementary Table S2: Top 10 differentially abundant proteins in human milk during later lactation.

Protein Groups	Genes	Protein Descriptions	Qvalue	Ratio
P05109	S100A8	Protein S100-A8	3.29E-02	5.61
P06702	S100A9	Protein S100-A9	4.07E-02	5.15
P06703	S100A6	Protein S100-A6	1.76E-03	2.71
P13796	LCP1	Plastin-2	1.71E-02	2.49
O00592	PODXL	Podocalyxin	4.84E-02	2.10
A0A075B6I0	IGLV8-61	Immunoglobulin lambda variable 8-61	3.49E-02	1.91
Q9UNW1	MINPP1	Multiple inositol polyphosphate phosphatase 1	5.95E-03	0.65
Q6WN34	CHRDL2	Chordin-like protein 2	1.43E-02	0.63
P01861	IGHG4	Immunoglobulin heavy constant gamma 4	1.08E-02	0.61
P04114	APOB	Apolipoprotein B-100	1.43E-04	0.60

Supplementary Figure S1: Protein intensities distribution in DIA-based human milk proteome quantitation.

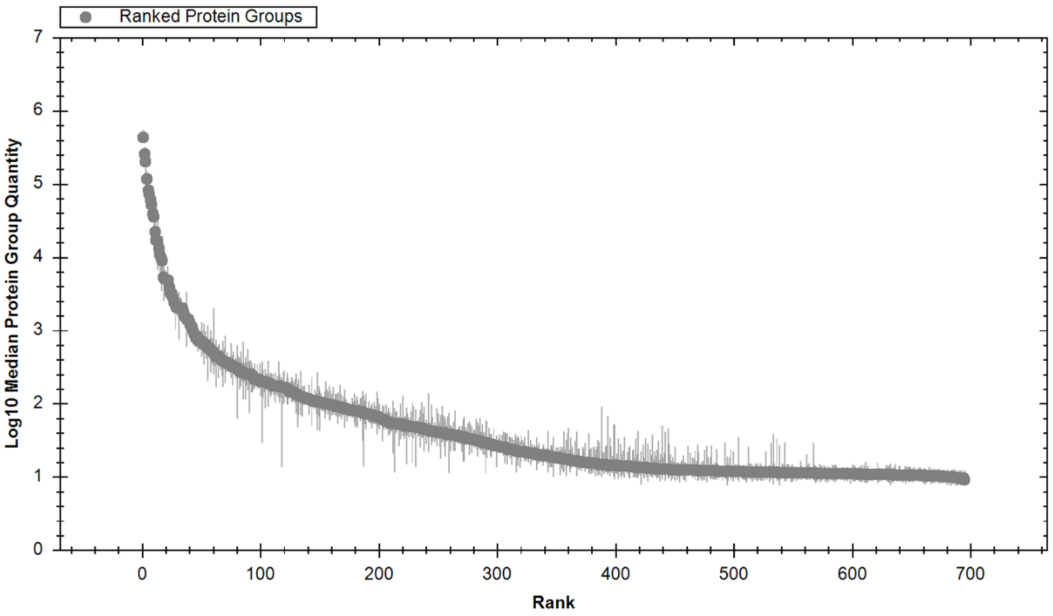

UniProt Id	Protein Description	Gene	Log10_MedianQuantity
P02788	Lactotransferrin	LTF	5.64
P00709	Alpha-lactalbumin	LALBA	5.31
P10451	Osteopontin	SPP1	5.07
P01876	Immunoglobulin heavy constant alpha 1	IGHA1	4.91
P01833	Polymeric immunoglobulin receptor	PIGR	4.87
P0DOX2	Immunoglobulin alpha-2 heavy chain	PIGR	4.78
P19835	Bile salt-activated lipase	CEL	4.71
P02768;	Albumin	ALB	4.60
P01591	Immunoglobulin J chain	JCHAIN	4.55
P10909	Clusterin	CLU	4.34

The dynamic range of the quantitatively measured proteins spanned 4.5 orders of magnitude (a typical value for this type of biological material obtained using the DIA method), with lactotransferrin, lactalbumin, albumin, and immunoglobulins among the top 10 most abundant proteins.
